# 

**Published:** 1895-07-13

**Authors:** 


					A.r>3VJi^U i i i-cnxTzn cmrrcixTrc dcjtt
CADBURY S ^ u ? OADBURYS
'OfaVi^T . ^JMk JMh "The Typical Cocoa of English Manufacture -
absolute purity and freedom ho?jj^)):waiic Absolutely Pure."? The Analyst.
Ft H ?
^ 'SSlff
swim
"^^S^CLBscotv Western Infirmary TivTril1 mTTTrtpriwpp1
No. 459 Yol XVin WITH EXTRA NURSING SUPPLEMENT. Per Annum, 10s.6d., port free. '

				

